# Histamine H_1_ receptor antagonists enhance the efficacy of antibacterials against *Escherichia coli*

**DOI:** 10.1186/s12917-019-1797-9

**Published:** 2019-02-11

**Authors:** G. G. Bruer, P. Hagedorn, M. Kietzmann, A. F. Tohamy, V. Filor, E. Schultz, S. Mielke-Kuschow, J. Meissner

**Affiliations:** 1 Department of Pharmacology, Toxicology and Pharmacy, University of Veterinary Medicine Hannover Foundation, Hannover, Germany; 2 Department of Toxicology and Forensic Medicine, Faculty of Veterinary Medicine, Cairo University, Giza, Egypt

**Keywords:** *Escherichia coli*, Antihistamines, Antibacterials, Checkerboard

## Abstract

**Background:**

H_1_ receptor antagonists are commonly used for the treatment of allergic diseases. The aim of this study was to find out, if antihistaminic compounds like mepyramine have the ability to influence the activity of antibacterials. Therefore, the checkerboard method was chosen to detect these possible effects in vitro. Studies were performed with two different *Escherichia coli (E. coli)* strains as test microbes, treated with antibacterials in combination with mepyramine.

**Results:**

The minimum inhibitory concentration (MIC) of *E. coli* ATCC® 25922™ and *E. coli* PIG 01 was reduced by combinations of the tested antibacterials with mepyramine**.**

**Conclusions:**

These results have to be confirmed in vivo, before the use of antihistamines should be considered as potential way to minimize the amount of used antibacterials for treatment of *E. coli* infections.

## Background

As mentioned by World Health Organization, BVL (Federal Office of Consumer Protection and Food Safety, Germany) and various other governmental and non-governmental organizations the number of infections caused by resistant bacteria is increasing in recent years in human beings and animals. For a targeted reduction of resistance new treatment options of infections are required. Brennan-Krohn et al. [1] showed synergistic effects of combinations of minocycline and colistin as well as minocycline and meropenem. In addition, Maier et al. [2] discovered antibacterial effects of various non antibiotic compounds, for example loratadine. El-Banna et al. [3] already described the modulation of antibiotic efficacy by antihistaminic drugs against *Klebsiella pneumoniae*.

It is also known that various bacteria (for example *Lactobacillus reuteri* and *Escherichia coli*) produce histamine under defined circumstances [4, 5], although the biological significance remains unclear. This mechanism leads to the hypothesis of the described study, that antihistamines potentiate the efficacy of antibacterials. Therefore, an investigation of interactions between antibacterial agents and histamine H1 receptor antagonists on the growth of *E. coli* was performed. *E. coli* is commonly found in the lower intestine of numerous animals and human beings as part of the commensal microbiota [6]. Two different *E. coli* strains were used. The first strain, *E. coli* ATCC® 25922™ represents a reference strain for antimicrobial susceptibility testing and is sensitive for enrofloxacin. The second strain (*E. coli* PIG 01) was isolated from pigs in own experiments and is resistant to enrofloxacin. El-Nakeeb et al. [7] already described growth-inhibiting effects on bacteria for H_1_ antihistaminic compounds like mepyramine, which represents a first generation H_1_ antihistamine [8].

Eight conventionally used antibacterials in veterinary medicine including amoxicillin, sulfadiazine/trimethoprim, tetracycline, colistin, enrofloxacin, florfenicol, gentamicin and kanamycin were tested in combination with mepyramine in vitro (checkerboard method).

## Results

Compared with the MIC of the compounds alone the MIC values of amoxicillin (penicillins), sulfadiazine combined with trimethoprim (trimethoprim-potentiated sulfonamides), colistin (polymyxins), enrofloxacin (fluoroquinolones), tetracycline (tetracyclines) and florfenicol were lower when combined with mepyramine (Table 1). For the combination of gentamicin (aminoglycosides) with mepyramine no interaction was found. Therefore, another aminoglycoside (kanamycin) was tested, which also showed no interaction. To exclude possible pH effects, pH changes were measured at 0 and 24 h of incubation for the combination of enrofloxacin and mepyramine. No pH changes were found.

**Table 1:** MIC of antibacterials in combination with mepyramine for *E. coli* ATCC® 25922™ and *E. coli* PIG 01

Combination with MEP	MIC (μg/ml)				DRI Median	DRI Range
	Alone		Combined			
	Median	Range	Median	Range		
*E. coli* ATCC® 25922™						
AMX	1.5	1.5–2	1^*^	1.0–1.5	1.5	1.3–1.5
SDZ/TMP	2	1–4	1^**^	0.3–1	3	2–8
TET	0.5	0.5–0.5	0.3^*^	0.3–0.5	2	1–2
CST	8	8–8	1^**^	1–2	8	4–8
ENR	0.01	0.01–0.01	0.005^**^	0.005–0.005	2	2–2
FFC	8	8–16	2^**^	2–4	4	4–4
GEN	0.3	0.3–0.5	0.3	0.3–0.5	1	1–1
KA	4	4.0–0.5	4	0.5–0.5	1	1–1
*E. coli* PIG 01						
AMX	1.5	1–3	1.3	0.8–2	1.5	1–1.5
SDZ/TMP	2	2–4	2	1–2	1.5	1–2
TET	1	1–4	0.5^*^	0.5–1	2	2–4
CST	2	2–4	1.5	1–2	2	1–4
ENR	16	16–16	8	8–16	2	1–2
FFC	8	8–32	2^**^	2–4	4	4–8
GEN	0.5	4–4	0.5	4–4	1	1–1
KAN	8	4–8	8	4–8	1	1–1

Data are expressed as median and range (*n* = 6)

Statistical significances were assessed between MIC alone and combined

*AMX* Amoxicillin, *SDZ/TMP* Sulfadiazine/trimethoprim, *TET* Tetracycline, *CST* Colistin, *ENR* Enrofloxacin, *FFC* Florfenicol, *GEN* Gentamicin, *KAN* Kanamycin, *MEP* Mepyramine, *MIC* Minimum inhibitory concentration, *DRI* Dose reduction index (MIC_alone_ / MIC_combined_)

^*^Significantly different (*p* < 0.05), ^**^Significantly different (*p* < 0.01)

**Fig. 1:**
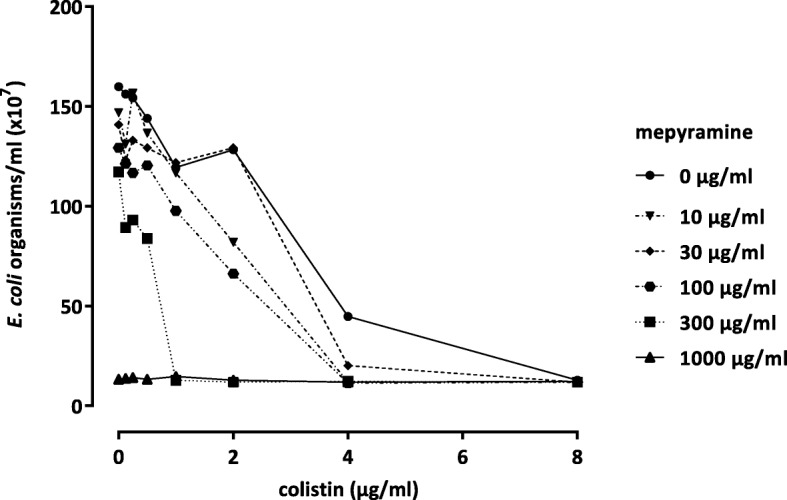
Representative example for the result of one checkerboard experiment with colistin and mepyramine. The lines show the growth of *E. coli* ATCC® 25922™ after 24 h depending on different colistin and mepyramine concentrations in combination. For colistin in combination with 300 μg/ml mepyramine the MIC of *E. coli* ATCC® 25922™ was reduced from 8.0 to 1.0 μg/ml

The highest dose reduction index (DRI) was calculated for colistin and florfenicol. For colistin in combination with mepyramine the MIC of *E. coli* ATCC® 25922™ was reduced from 8.0 to 1.0 μg/ml (Fig. 1). In addition, for florfenicol the MIC of *E. coli* ATCC® 25922™ and *E. coli* PIG 01 was decreased from 8.0 to 2.0 μg/ml.

To exclude cytotoxic effects of mepyramine on the growth of *E. coli*, the ratio of viable and dead bacteria cells were determined. The positive control showed 98% viable cells and the negative control 100% dead cells. All mepyramine suspended samples of *E. coli* exhibited 100% viable cells.

## Discussion

The antibacterial effect of antihistamines described by El-Nakeeb et al. [7] was confirmed in the present study for concentrations of 1000 μg/ml mepyramine. Additionally, it was found that mepyramine was able to enhance the antibacterial effects of amoxicillin, sulfadiazine combined with trimethoprim, colistin, enrofloxacin, tetracycline and florfenicol against *E. coli* ATCC® 25922™ and *E. coli* PIG 01. This is not a cytotoxic effect as shown in the present study due to 100% living cells after mepyramine incubation for 24 h in different concentrations.

Possible mechanisms for the enhanced efficacy of antibacterials in presence of antihistamines were already discussed by El-Banna et al. [3], but the exact effect seems to be unclear.

El-Banna et al. [3] argues that the enhanced efficacy should be caused by an inhibition of bacterial efflux pumps by antihistamines. They also mentioned an inhibition of biofilm formation for the antihistamine promethazine by inhibiting quorum sensing. Another idea is based on the main structural feature of antihistamines, a tertiary amino group and a lipophilic aromatic moiety, hence they possess certain surfactant-like characters [9]. Possible properties like this might cause alterations of biological membrane permeability [10, 11] which could negatively affect the bacteria. Hagmar et al. [12] showed that antihistamines could influence bacterial cells by binding to the minor groove of the bacterial DNA and intercalating between the base pairs. It can be hypothesized that bacteria like *E. coli* produce histamine under stress conditions possibly as a protection against environmental factors. Postulating that this effect is mediated via bacterial histamine receptors, histamine receptor antagonists could interact with this mechanism. Therefore, the efficacy of antibacterial agents should be enhanced. These assumptions have to be proven by further studies.

Antihistamines have been classified into two generations, the first-generation and second-generation and moreover in various chemical groups: the ethanolamines, ethylenediamines, alkylamines, piperazines, piperidines, phenothiazines and others [8]. The results in this study for the ethylenediamine mepyramine were compared in single experiments with the ethanolamine diphenhydramine and for the second generation antihistaminic drug the piperazine cetirizine and for others azelastine. In the current study, an antibacterial dose reduction was confirmed only for a combination with the first generation antihistamines, which is in contrast to El-Banna et al. [3], who found similar effects for different antibiotics in combination also with cetirizine on the growth of *Klebsiella pneumoniae*.

Second-generation H_1_ antihistamines are newer drugs that are much more selective for peripheral H_1_ receptors [13]. It could be imagined that bacteria may have unspecific binding sides for antihistamines, therefore cetirizine and azelastine are not able to effect because of their specificity. Based on the present study this difference in chemical structure may be the reason for the shown enhanced efficacy only in combination with the first generation antihistamines. Furthermore, the combination with mepyramine enhanced the efficacy for six of the used antibacterials. No effects on the aminoglycosides gentamicin and kanamycin in combination with mepyramine were shown, which is in contrast to the study results of El-Banna et al. [3], where an enhanced efficacy was shown for the combination of cetirizine and diphenhydramine on the growth of *Klebsiella pneumoniae*, as well.

These in vitro findings need to be confirmed in vivo. Besides, more research is required to answer the raised questions and to get a better idea of how bacteria, histamine and antihistamines are interacting.

## Conclusion

The combined use of antihistamines and antibacterials might be a potential option to treat infectious diseases in future and to reduce the absolute amount of antibacterials used therapeutically.

## Methods

### Bacterial culture

Two different *E. coli* strains were used. The first strain, *Escherichia coli* ATCC® 25922™ (American Type Culture Collection, Manassas, VA, USA) represents a reference strain for antimicrobial susceptibility testing and is sensitive for enrofloxacin (MIC ≤ 0.03 μg/ml). A second strain (*E. coli* PIG 01) was isolated from pigs in own experiments and exhibits an MIC for enrofloxacin of ≥ 16 μg/ml.

### Agents

Mepyramine maleate was obtained from Tocris Bioscience (Bristol, UK) and Sigma-Aldrich (St. Louis, MO, USA). Cetirizine dihydrochloride, azelastine hydrochloride, diphenhydramine hydrochloride, amoxicillin, sulfadiazine sodium salt, trimethoprim, enrofloxacin, colistin sulfate salt, gentamicin sulfate and kanamycin sulfate were purchased from Sigma-Aldrich (St. Louis, MO, USA). Tetracycline hydrochloride was obtained from Carl Roth (Karlsruhe, Germany) and florfenicol from Cayman Chemical Company (Ann Arbor, MI, USA). All agents were directly diluted in M9 minimal medium (described below), except florfenicol, which was first dissolved in 10 μl dimethyl sulfoxide (DMSO), and enrofloxacin by adding 5% of 1 N sodium hydroxide solution. The trimethoprim-potentiated sulfonamides were used at a ratio of 19:1 (sulfadiazine:trimethoprim) stock solution dissolved with 0.5 ml of DMSO in 20 ml M9 minimal medium.

### Medium

M9 minimal medium was used for the cultivation of *E. coli* strains. This buffered minimal microbial medium is composed of 5 g glucose, 6 g disodium hydrogen phosphate, 3 g potassium dihydrogen phosphate, 1 g ammonium chloride, 0.5 g sodium chloride, 120 mg magnesium sulfate, 10 mg calcium chloride and 20 mg thiamine hydrochloride in one liter double distilled water.

### Checkerboard method

**Fig. 2:**
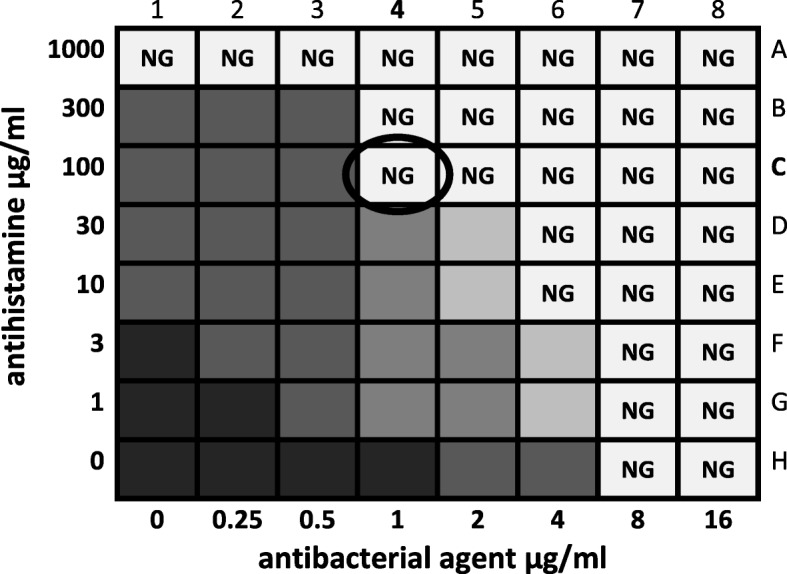
Schematic explanation of a checkerboard experiment in a 96-multiwell-plate. This example shows an enhanced efficacy of two drug combinations. The antibacterial agent is applied along the columns and the antihistamine along the rows, both in increasing concentrations. The decrease of bacterial growth is presented schematically by fading shades of gray. “NG” indicates no growth of bacteria. In this example, the effect of the antibacterial agent is enhanced in well C4 (circled)

To identify an enhanced efficacy of an antibacterial agent in combination with a non-antibacterial active agent, the in vitro checkerboard method was used [14]. The *E. coli* strains were subcultured on 7% columbia sheep blood agar plates (Thermo Fisher Scientific, Waltham, MA, USA) for 24 h at 37 °C. An *E. coli* inoculum was suspended in M9 minimal medium and adjusted to a 0.5 McFarland turbidity standard correlating to 1.5 × 10^8^ CFU/ml (colony-forming unit). The agents were diluted with M9 minimal medium. The checkerboard tests were carried out in 96-multiwell-plates (Greiner, Kremsmuenster, Austria) containing a total volume of 200 μl per well including bacteria suspension. Controls were filled with medium, antihistaminic or antibacterial agent and bacteria suspension. The H_1_ receptor antagonist was serially diluted along the rows in logarithmic increasing concentrations from 0 up to 1000 μg/ml, various antibacterial agents along the columns starting at zero and ending at two times MIC (Fig. 2).

Then each well was filled with an unique combination of the two substances (for example well C4 in Fig. 2 with 50 μl of 100 μg/ml mepyramine and 50 μl of 1 μg/ml colistin). Finally, M9 minimal medium containing 1.5 × 10^8^ CFU/ml was added to the wells. To exclude effects caused by pH changes the pH was evaluated at time points 0 and 24 h after incubation for the combination of enrofloxacin and mepyramine in every well of the 96-multiwell-plate with pH paper (range 1–14, Carl Roth, Karlsruhe, Germany).

Bacterial growth was compared after 24 h of incubation at 37 °C by measuring the absorbance with a microplate reader at 570 nm (MRX Microplate Reader, Dynatech Laboratories, Channel Islands, GB). Each single study was performed six times. The concentration of the first well without visible bacterial growth due to the combination of two agents was used to determine the MIC_combined_ and was compared with the respective MIC_alone_. A dose reduction index (DRI) was calculated according to Chou [15] by means of:

$$ \mathrm{DRI}=\frac{\ {\mathrm{MIC}}_{\mathrm{alone}}}{{\mathrm{MIC}}_{\mathrm{combined}}} $$

### Cytotoxic effects of the test compounds

To determine the amount of viable and dead bacteria under mepyramine stress conditions (30, 100 and 1000 μg/ml mepyramine, incubation time 24 h at 37 °C) the LIVE/DEAD™ BacLight™ Bacterial Viability Kit (Thermo Fisher Scientific, Waltham, MA, USA) was used according to manufactures manual. The assay contains two fluorescent nucleic acid stains (SYTO 9 and propidium iodide). Bacteria with intact membranes (living bacteria) are stained fluorescent green, whereas cells with damaged membranes (dead bacteria) are stained fluorescent red. Bacteria cultures without adding any agent served as positive control. Bacteria with addition of isopropylalcohol (70%, Honeywell, Morristown, NJ, USA) were used as negative control. The stained bacterial suspension was analysed using fluorescence microscopy (Leica, Wetzlar, Germany) by counting the stained bacterial colonies in two visual fields (100 x magnification) at 490 and 546 nm [16, 17].

### Statistical analysis

Statistical significances of differences between MIC values were assessed using the Mann-Whitney U-test (GraphPad Prism version 7.04, GraphPad Software, Inc., La Jolla, CA, USA). Statistical significance was set at a *p* value < 0.05. Data given in the text and table are presented as median and range.

## Abbreviations

°C: Degree Celsius
AMX: Amoxicillin
ATCC: American Type Culture Collection
BVL: Federal Office of Consumer Protection and Food Safety
CFU: Colony-forming unit
CST: Colistin
DMSO: Dimethyl sulfoxide
DRI: Dose reduction index
*E. coli*: *Escherichia coli*
ENR: Enrofloxacin
FFC: Florfenicol
g: Grams
GEN: Gentamicin
KAN: Kanamycin
MEP: Mepyramine
mg: Milligrams
MIC: Minimum inhibitory concentration
ml: Millilitre
NG: No growth of bacteria
nm: Nanometre
SDZ/TMP: Sulfadiazine/trimethoprim
TET: Tetracycline
μg: Micrograms
μl: Microliter

## References

[1] Brennan-Krohn T, Truelson KA, Smith KP, Kirby JE (2017). Screening for synergistic activity of antimicrobial combinations against carbapenem-resistant Enterobacteriaceae using inkjet printer-based technology. J Antimicrob Chemother.

[2] Maier L, Pruteanu M, Kuhn M, Zeller G, Telzerow A, Anderson EE, Brochado AR, Fernandez KC, Dose H, Mori H (2018). Extensive impact of non-antibiotic drugs on human gut bacteria. Nature.

[3] El-Banna T, Sonbol F, El-Aziz A, Al-Fakharany O (2016). Modulation of antibiotic efficacy against Klebsiella pneumoniae by antihistaminic drugs. J Med Microb Diagn.

[4] Thomas CM, Hong T, van Pijkeren JP, Hemarajata P, Trinh DV, Hu W, Britton RA, Kalkum M, Versalovic J (2012). Histamine derived from probiotic lactobacillus reuteri suppresses TNF via modulation of PKA and ERK signaling. PLoS One.

[5] Barcik W, Pugin B, Westermann P, Perez NR, Ferstl R, Wawrzyniak M, Smolinska S, Jutel M, Hessel EM, Michalovich D (2016). Histamine-secreting microbes are increased in the gut of adult asthma patients. J Allergy Clin Immunol.

[6] Allocati N, Masulli M, Alexeyev MF, Di Ilio C (2013). Escherichia coli in Europe: an overview. Int J Environ Res Public Health.

[7] El-Nakeeb MA, Abou-Shleib HM, Khalil AM, Omar HG, El-Halfawy OM (2011). In vitro antibacterial activity of some antihistaminics belonging to different groups against multi-drug resistant clinical isolates. Braz J Microbiol.

[8] Simons FER, Simons KJ (2008). H(1)antihistamines: current status and future directions. World Allergy Organ J.

[9] Attwood D, Florence AT. Physicochemical principles of pharmacy. 6th ed. London: Pharmaceutical Press; 2016. p. 193-246.

[10] Guth PS, Spirtes MA. The Phenothiazinetranquilizers: Biochemical and Biophysical Actions. Int Rev of Neurobiol. 1964;7:231-278. 10.1016/s0074-7742(08)60269-x 14289332

[11] Molnar J, Kiraly J, Mandi Y (1975). The antibacterial action and R-factor-inhibiting activity by chlorpromazine. Experientia.

[12] Hagmar P, Pierrou S, Nielsen P, Norden B, Kubista M (1992). Ionic strength dependence of the binding of methylene blue to chromatin and calf thymus DNA. J Biomol Struct Dyn.

[13] Camelo-Nunes IC (2006). New antihistamines: a critical view. J Pediatr.

[14] Zuo GY, Yang CX, Han J, Li YQ, Wang GC (2018). Synergism of prenylflavonoids from Morus alba root bark against clinical MRSA isolates. Phytomedicine.

[15] Chou T-C (2006). Theoretical basis, experimental design, and computerized simulation of synergism and antagonism in drug combination studies. Pharmacol Rev.

[16] Boulos L, Prevost M, Barbeau B, Coallier J, Desjardins R (1999). LIVE/DEAD BacLight : application of a new rapid staining method for direct enumeration of viable and total bacteria in drinking water. J Microbiol Methods.

[17] Stiefel P, Schmidt-Emrich S, Maniura-Weber K, Ren Q (2015). Critical aspects of using bacterial cell viability assays with the fluorophores SYTO9 and propidium iodide. BMC Microbiol.

